# Professional protocol and rational outlook of dentists towards SDF

**DOI:** 10.6026/97320630018547

**Published:** 2022-06-30

**Authors:** Zohra Jabin, V Vishnu Priya, Iffat Nasim

**Affiliations:** 1 Saveetha Dental College, Saveetha Institute of Medical and Technical Sciences, Saveetha University, Chennai, India

**Keywords:** Prevention, silver diamine fluoride, dental caries, cariostatic agents, dentists, surveys, questionnaires

## Abstract

SDF has gained immense popularity worldwide in the recent years due to its dual performance brought about by inhibiting bacterial growth as well as promoting the remineralisation of dental hard tissues. This study aims to evaluate the knowledge and attitude among dentists towards Silver Diamine Fluoride (SDF). The present study is an online survey which was designed using Google forms, to gather information about the knowledge and professional protocol followed by dentists for SDF use in their respective operatories. Written informed consent was obtained from the participants after explaining to them the purpose of the study. The detailed questionnaire comprised of two sections. First section comprised of 15 questions which inquired about SDF knowledge and protocols followed for its use by dentists. Second section analyzed rational outlook of dentists towards SDF. Sample selection was done by simple random sampling and questionnaire Google link was circulated among 224 dentists. The mean age group of the participants is 33.82 ± 12 years. A statistically significant difference was found between the participant and the use of SDF in operatory, its application for performance in cavitated or non cavitated lesions, application intervals and the potential problems associated with SDF use. A majority of dentist (62.5%) knew that 38% concentration of SDF to be used among the children which is statistically significant. (p value ≤0.05).A lack of self-reported knowledge was most frequently reported concerning the use and application of SDF among patients to arrest carious lesions in primary and permanent teeth in a dental setting. Thus further studies can be of excellent utility especially for whole community with limited resources instead of using costly preventive strategies.

## Background:

Dental caries has a profound effect on general as well as oral health. Untreated dental caries may progress to severe dental infection and oro-facial pain compromising the ability to chew and eat. This may eventually lead to a functional impairment which affects an individual's growth and overall development. Early childhood caries (ECC) has emerged as a problem of concern globally [1]. ECC can begin early in life, progresses rapidly in those who are at high risk, and may often sometimes go untreated [2-3]. The consequences can affect the immediate and long-term quality of life of the child and family, and can have a significant social and economic consequence as well [4]. SDF has gained immense popularity worldwide in the recent years. It has drawn increased attention due to its dual performance brought about by inhibiting bacterial growth as well as promoting the remineralisation of dental hard tissues. Silver and fluoride constitute as its main components, which act synergistically and makes this solution a potent caries arresting/preventing agent unlike other topical fluoride agents. SDF offers non invasive treatment option and gained popularity due to its due to the shift from invasive to preventive and conservative approaches for caries management owing to the better understanding of caries process overtime. The ease of use makes it desirable, especially for developing low income countries where population encounter low accessibility and utilization of basic dental procedures and struggle to combat the economic burden placed on them due to high costs of caries treatment. Being cost-effective and having a simple application procedure, SDF can be advocated for use as an appropriate intervention for community settings [5-6]. SDF use is advocated strongly in children as it is challenging to perform long term multiple visit treatment due to their behavioral issues and lack of co-operation. Therefore, SDF has a promising effect on active inhibition of dental caries and its use has been popularized but still there is a lack in the knowledge and awareness about its application protocol among health care providers. Thus, the present survey was planned to assess the professional protocol and rational outlook followed by practicing dentists towards the use of SDF.

## Materials and methods:

A questionnaire based observational online cross-sectional survey was conducted to gather information about the knowledge and professional protocol followed by dentists for SDF use in their respective operatories. Ethical approval for performing the survey was obtained from the Institutional review board ((STP/SDMDS2015PED42D)) prior to the conduct of the study. The purpose of study was explained by telephonic conversation to them and it was informed that their participation is completely voluntary. A pilot study was conducted prior to check for the feasibility of the questionnaire and for its validity and reliability. A total of 240 dentists who were engaged in clinical postings and were willing to participate were included in the study. Out of 240 subjects approached for the study only 224 agreed to be the part of the study. Unwilling participants were excluded from the study. The mode of collecting the responses was online mode for which a questionnaire was created on Google form. A set of self administered and validated questionnaires were shared through online mode among the study subjects. The questionnaire had short answer option for filling demographic information like name, age, qualification and designation etc. along with multiple choice fill in response questions for obtaining information about SDF knowledge and protocols. Demographic information like name, age, qualification, designation etc. was obtained from each participant. The detailed questionnaire was divided into two sections. The first section comprised of 15 questions which inquired about SDF knowledge and protocols followed for its use by the dentists while the second section analyzed the rational outlook of dentists towards SDF. Survey reports were kept anonymous and participant's confidentiality was assured. Data were entered into Microsoft Excel and differences between the groups were checked using SPSS (Statistical Package for Social Sciences) Version 16.0; IBM SPSS Inc., Chicago, IL, USA. The data were subjected to quantitative analysis. Chi-square test and Shapiro-wilk test was used to test the significant difference between the three groups of professionals (P ≤ 0.05) and assess the normality of the data. The level of significance and confidence interval were 5% and 95% respectively.

## Results:

A total of 224 general dentists, and specialists responded to the questionnaire. The mean age group of the participants is 33.82 ± 12 years. All age group dentists responded to the survey 42.9% of study participants were between the ages 20-30 years, 33.9% were of 31- 40 years, 19.6 were of 41-50 years, and 3.6% were >50 years. The demographic characteristics of participants were tabulated in Table 1(see PDF).

Most dentists didn't perform caries risk assessment prior to case selection for topical SDF application (71.4%). Only very few used stated the use of SDF only as a caries preventive agent on carious (8.9%) as well as sound teeth (8.9%) whereas a majority of them stated the use of SDF for caries arresting agent on carious teeth (67.9%). Although, some dentists does SDF application in patients having only high plaque score but their number was very less (3.6%).Very few were aware of SDF action on non cavitated lesions(12.5%) and about half of them used it in patients with both cavitated and non cavitated lesions (50%). A few dentists advocated SDF application in primary teeth (30.4%), however most respondents practiced SDF application in both dentition (67.9%). Almost half (58.9%) of the participants placed restorative material post SDF and rest preferred the crown placement over restoration (41.1%). It was observed that there was a lack in clarity regarding the application interval among dentist and about 53.6% of dentists preferred biannually application of SDF. Majority of the participants preferred hand excavation for caries removal (75% ) than drilling, agreed to take a consent prior to application of SDF (51.8%), were not aware of the time gap preferred between SDF and KI application (44.9%), applied petroleum jelly for stain prevention on surrounding tissues (66.1%). It is recommended that prior caries removal is not required for SDF application but results suggested that many were not aware about it and made it a point to excavate caries prior to applying SDF. Black staining is the potential problem encountered with SDF use by majority of dentist in their operatory (60.7%). A statistically significant difference was found between the participant and the use of SDF in operatory, its application for performance in cavitated or non cavitated lesions, application intervals and the potential problems associated with SDF use (p value ≤0.05) (Table 2 - see PDF). A total of 46.4% dentist agreed that SDF can be a good treatment for dentally anxious children and only 19.6%dentist preferred SDF over conventional treatment, 48.2% occasionally took radiographs prior to SDF application. A majority of dentist (62.5%) knew that 38% concentration of SDF to be used among the children which is statistically significant (p value ≤0.05) (Table 3 - see PDF).

## Discussion:

SDF has an ultimate goal to treat tooth hypersensitivity and arrest cavitated carious lesions which may be accomplished by painting the cavitated lesion with SDF liquid without removing any infected soft dentin [7,8]. AAPD has recently released clinical practice guidelines for SDF and also advocated its use for caries management. SDF treatment can prove a boon for the patients who lack an immediate access to traditional restorative treatment [9]. In the present study half of respondents (50%) agreed that SDF can be used to arrest both cavitated and non cavitated lesions in dentin. However 37.5% of them agreed that SDF should be applied only for cavitated lesions which is well supported by existing evidence [7, 8,10]. Further, only 12.5% dentists agreed that SDF can be used to treat only non-cavitated lesions in enamel but there is very limited clinical evidence supporting that treatment. Around 57.1% respondents were in favour of placing SDF prior to caries removal, and 42% favored placing SDF post caries removal. Although use of SDF is indicated prior to restoring teeth but the literature lacks sufficient evidence in this regard. The available clinical studies concerning the role of SDF in prevention of secondary caries are limited and have had contrary conclusions [11, 12] despite a positive role suggested by vitro datas.[13] Thus, there is an urge of further research to explore these aspects. The present study showed that 60.7% dentist encountered the adverse effects of SDF. It leads to black discoloration of carious lesion, which is mainly due to the precipitation of silver phosphate layer after SDF application.[9] To overcome this drawback and enhance the clinical use of SDF, a study by Ngo et al. [14] have proposed a method to overcome the staining due to SDF has suggested the application of a layer of potassium iodide (KI) over the initial layer of SDF to overcome staining which then reacts with the free silver ions in SDF and prevents the formation of silver phosphate precipitate. Similar findings were observed in studies by Nguyen V and Miller MB et al. [15- 16]. Nearly about 67.9% participants agreed that SDF will be applied to both primary and permanent teeth. Besides only 1.8% agreed to the fact that SDF will be applied only in permanent teeth which is comparable to a study done by M Zakirulla et al. where participants disagreed to the statement that SDF is found in permanent teeth in comparison with primary teeth. [17] The reason might be that SDF darkens the tooth leading to a problem of aesthetics and also it is better to recognize in a short-term tooth thus proving that SDF works more effectively in primary than in permanent teeth [18]. A recent study found that parents accepted SDF treatment depending on whether it was a posterior lesion (68%) or an anterior lesion (30%) [9]. There is a higher inclination of parents to the use of SDF in the posterior tooth when compared to anterior teeth which again might be due to the problem of aesthetics. [9] Around 53.6% approved biannual application of SDF in their clinics has been better than once annually applied SDF. It is stated by a study by Yee et al that biannual application of 38% SDF solution could arrest caries by 84% and the dose response ratio of 38% SDF is better than 12% SDF solution. The study also further discovered that arrested cavitated lesions portion reduced over an interval of two years following a single initial program thus initiating a need of re-application with passage of time. [19] Interestingly majority of dentist were already aware of the fact that 38% of SDF application is most reliable for arresting dentine caries which is comparable to a study done by Chibinski et al. [10]. Majority of dentist agreed to the fact that SDF can be most commonly used among children with behavioral problems as well as among anxious patients since it is a simple and low-effective technique that does not need child cooperation. Another study by Nelson et al. study among pediatric dentistry program administrators found that SDF can be indicated for treating patients with behavioral issues and for medically fragile patients.[20] The literature stated that the dentists should aware parents about the easy application, child -friendly tactic of SDF and even it helps to avoid treatments with physical restraint or to treat under general anesthesia.[21] This suggestion is based on the fact that negative feelings of the parents about tooth -staining decreased when they understood simplicity of SDF technique. Thus further studies in this regard could improvise the overall impact as well as pros and cons of SDF approach that can be of excellent utility and cost effective especially for the whole community with limited resources instead of using costly preventive strategies.

## Conclusion:

A lack of self-reported knowledge was most frequently reported concerning the use and application of SDF among patients to arrest carious lesions in primary and permanent teeth in a dental setting. Thus educational efforts are needed to increase knowledge about the proper use, benefits, and limitations of SDF among the dental clinicians.. Increasing SDF educational efforts might therefore result in increased utilization of this new approach to treating cavitated caries lesions, especially in children. Thus, further research must focus on SDF educational interventions in more detail with a larger sample population. More information and emphasis should also be given to continuing education and online web-based resources in further studies.

## References

[1] Livny A (2007). BMC Public Health..

[2] Grindefjord M (1995). Caries Res..

[3] Weinstein P (1994). ASDC J Dent Child..

[5] Clemens J (2018). J. Public Health Dent..

[6] Lo EC (2001). J Dent Res..

[7] Gao SS (2016). Clin Trans Res..

[8] Chu CH (2002). J Dent Res.

[9] Crystal YO,  Niederman R (2016). Pediatr Dent..

[10] Chibinski AC (2017). Caries Res.

[11] Zhao IS (2017). J Dent.

[12] McDonald SP,  Sheiham A (1994). Int Dent J.

[13] Mei ML (2016). Int Dent J.

[14] Knight GM (2006). Aust Dent J..

[15] Zhao IS (2017). Int J Mol Sci..

[16] Miller MB (2016). American journal of dentistry..

[17] Zakirulla M (2021). J Res Med Dent Sci..

[18] Llodra JC (2005). J Dent Res.

[19] Yee R (2009). J Dent Res.

[20] Nelson T (2016). Pediatr Dent.

[21] Clemens J (2018). J Public Health Dent.

